# Green Synthesis of a Molecularly Imprinted Polymer Based on a Novel Thiophene-Derivative for Electrochemical Sensing

**DOI:** 10.3390/molecules29071632

**Published:** 2024-04-05

**Authors:** Francesco Gagliani, Tiziano Di Giulio, Sara Grecchi, Tiziana Benincori, Serena Arnaboldi, Cosimino Malitesta, Elisabetta Mazzotta

**Affiliations:** 1 Laboratorio di Chimica Analitica, Dipartimento di Scienze e Tecnologie Biologiche e Ambientali (Di.S.Te.B.A.), Università del Salento, Via Monteroni, 73100 Lecce, Italy; francesco.gagliani@unisalento.it (F.G.); cosimino.malitesta@unisalento.it (C.M.); 2 Dipartimento di Chimica, Università di Milano, Via Golgi 19, 20133 Milano, Italy; sara.grecchi@unimi.it (S.G.);; 3 Dipartimento di Scienza e Alta Tecnologia, Università degli Studi dell’Insubria, Via Valleggio 11, 22100 Como, Italy

**Keywords:** green chemistry, green synthesis, thiophene-derivative, ionic liquid, tyrosine, molecularly imprinted polymer, amperometric sensor, electrochemical sensor

## Abstract

An environmentally friendly and sustainable approach was adopted to produce a molecularly imprinted polymer (MIP) via electropolymerization, with remarkable electrochemical sensing properties, tested in tyrosine (tyr) detection. The 2,2′-bis(2,2′-bithiophene-5-yl)-3,3′-bithianaphtene (BT_2_-T_4_) was chosen as functional monomer and MIP electrosynthesis was carried out via cyclic voltammetry on low-volume (20 μL) screen-printed carbon electrodes (C-SPE) in ionic liquid 1-butyl-3-methylimidazolium bis(trifluoromethylsulfonyl)imide ((BMIM) TFSI). An easy and rapid washing treatment allowed us to obtain the resulting MIP film, directly used for tyr electrochemical detection, carried out amperometrically. The sensor showed a linear response in the concentration range of 15–200 μM, with LOD of 1.04 µM, LOQ of 3.17 μM and good performance in selectivity, stability, and reproducibility. Tyrosine amperometric detection was also carried out in human plasma, resulting in a satisfactory recovery estimation. The work represents the first use of BT_2_-T_4_ as a functional monomer for the production of a molecularly imprinted polymer, with a green approach afforded by using a few microliters of a room temperature ionic liquid as an alternative to common organic solvents on screen-printed carbon electrodes, resulting in a valuable system that meets the green chemistry guidelines, which is today an essential criterion in both research and application field.

## 1. Introduction

Molecularly imprinted polymers (MIPs) are synthetic receptors obtained from the polymerization in a proper solvent of functional monomers and cross-linking agents around the target molecule, thus serving as a template that, once removed, leaves complementary cavities able to rebind the analyte specifically [1,2,3,4]. The principle behind the molecular imprinting technology is to mimic the molecular recognition that naturally occurs in living systems, such as enzyme-substrate and antibody-antigen complexes [5]. However, unlike their biological counterparts, MIPs possess many advantages, such as robustness and stability, as well as easy and low-cost production processes [6].

Several methods can be used to produce imprinted polymers, for example, bulk polymerization, precipitation polymerization or emulsion polymerization [7], along with solid-phase approaches, today widely used for their synthesis in the form of nanoparticles. Although the MIP production processes may be very simple, in some cases, they may have an impact on operator health and environment. Indeed, not only can the manufacture of an MIP be dangerous, especially when hazardous reagents and solvents are used, but also their application and discarding can raise health and environmental concerns [8]. MIP production often requires the use of organic solvents for promoting monomer-template interactions with no detrimental effect from the solvent nature, being hydrogen and electrostatic bonding between monomer and template hindered in aqueous solutions, which are thus less suitable for the imprinted cavities formation [9]. Moreover, once the imprinting procedure is performed in an organic solvent, the subsequent rebinding step should preferentially be in the same media having the same interactions involved. However, organic solvents have a well-known dangerous impact on human health by affecting the respiratory tract and the cardiovascular system, skin and liver [10], as well as on the environment. As an example, volatile organic compounds (VOCs) can promote the formation of the so-called ground-level ozone, which reduces plant growth and photosynthesis ability [11,12].

Nowadays, the objective of the Sustainable Development Goals (SDGs) of the United Nations 2030 Agenda for Sustainable Development is to counteract the risk linked to the synthesis of a certain chemical product by adopting the concept of green chemistry, which aims to develop eco-friendly chemical procedures with improved human life and environmental quality [13]. Also, the perspective large-scale production of MIPs needs greener strategies that, as explained by the acronym GREENIFICATION, require minimal waste production and treatment, the use of renewable reagents, mild polymerization conditions and aqueous or at least greener solvents [8].

With this aim, some strategies have been implemented, for example, ultrasound- and microwave-assisted MIP synthesis. Svenson prepared an MIP for theophylline via sonication, obtaining a great template solubility as well as a rapid polymer formation at 35 °C [14], while Lamaoui et al. produced magnetic molecularly imprinted polymers for sulfamethoxazole with a microwave-assisted synthesis, requiring only 20 min to obtain polymers with a high imprinting factor [15]. Other MIP syntheses in aqueous solutions have been attempted. For example, Gheybalizadeh and Hejazi used the precipitation method to prepare magnetic molecularly imprinted polymers toward insulin by dissolving acrylamide, methacrylic acid and 2-dimethyl aminoethyl methacrylate as functional monomers in 0.1 M PBS, claiming a high imprinting factor as well as a great selectivity [16]. The use of organic solvents can be replaced by greener options such as supercritical carbon dioxide (scCO_2_), a non-toxic, non-flammable and inert medium that was used for the first time by Ye et al. to produce molecularly imprinted polymer nanoparticles for propranolol, showing binding abilities similar to the imprinted polymer produced in organic solvents [17]. Ionic liquids (ILs) represent another valid alternative able to improve the polymerization reaction rate, thanks to electrostatic interactions, hydrophobic interactions and hydrogen bonds between ILs and the other components of the system, affecting the radical reactivity [18]. ILs are defined as solvents totally constituted by a cation and an anion (Table 1), possessing several advantageous characteristics, such as stability and low vapor pressure, non-flammability as well as environmental safety, since some of them can be synthesized in harmless conditions and it may possible to purify the solvent after its use [19,20,21,22]. Due to their extraordinary features, ILs are currently used in a wide range of applications. In particular, the so-called room temperature ionic liquids (RTILs), which are liquid at ambient conditions, are now considered great and promising replacements for organic solvents, thanks to their range of solubility, miscibility, absence of volatility and especially the possibility to tune IL behavior by changing the cation/anion composition [23].

Interestingly, RTILs offer tremendous advantages from an electrochemical point of view, such as wide potential windows and, being totally constituted by ions, high conductivity, therefore eliminating the necessity of supporting electrolytes, unlike organic solvents [24]. These features led to a deep investigation of the ionic liquid roles in electrochemistry, highlighting that ILs can stabilize radical cations and anions produced by electrochemical oxidation or reduction of species such as amines or carbonyl compounds, respectively, therefore allowing to obtain high yields of the product [25]. What is more, ionic liquids have been extensively studied in the polymer chemistry field. For example, Qi et al. analyzed the electrochemical polymerization of aniline in 1-butyl-3-methyl-imidazolium tetrafluoroborate ((BMIM)BF_4_), obtaining a film that showed a great electrochemical response in acidic solution and outstanding catalysis abilities towards targets such as catechol and hydroquinone [26], while Sekiguchi et al. investigated the electropolymerization of pyrrole in a mixture of ionic liquid 1-ethyl-3-methylimidazolium trifluoromethane sulfonate (EMICF_3_SO_3_) and H_2_O, reporting not only an improved polymerization rate but also a film with greater electroconductivity than that produced in a mixture of the same ionic liquid with acetonitrile. Additionally, the authors claimed that it was possible to recover the medium by extracting pyrrole after reaction with chloroform without any decrease in its reactivity [27].

The distinct advantages of ionic liquids have been applied to molecularly imprinted polymers. The roles of ILs in molecular imprinting technology are nowadays widespread, mainly because they can be used as porogen [28] or cross-linking agents [29] and even functional monomers [30]. For instance, Booker et al. developed a *trans*-aconitic acid MIP in 1-butyl-3-methylimidazolium tetrafluoroborate (BMIM)BF_4_ and 1-butyl-3-methylimidazolium hexafluorophosphate (BMIM)PF_6_ under photochemical or thermal initiation reporting not only a higher solubility of the molecules involved in the reactions but also a reduced swelling degree of the polymers, that, according to the authors, resulted in enhanced selectivity [31]. A robust approach in imprinting technology combines the extraordinary electrochemical features of ionic liquids with advantages related to electropolymerization, that is, a simple and rapid approach to obtain a film with controlled thickness directly onto the electrode surface by selecting the amount of circulating charge [32]. An example of a molecularly imprinted polymer electrosynthesized in ionic liquid was reported by Pietrzyk et al., who developed a piezomicrogravimetric chemosensor for melamine detection by using cyclic voltammetry to copolymerize a bis(bithiophene)methane, decorated with a benzo-18-crown-6 substituent, with a thiophene-derivative as cross-linking agent around the target in trihexyl(tetradecyl)phosphonium tris(pentafluoroethyl)-trifluorophosphate ionic liquid. The results showed an improved chemosensor performance due to the presence of the cross-linking agent and ionic liquid, as well as a limit of detection of 5 nM and an excellent selectivity against melamine structural analogs [33]. Hu et al. gave an interesting contribution about the effect of ionic liquids as electrolytes in the electrosynthesis of a molecularly imprinted polymer to determine patulin by using two dummy templates to obtain different types of cavities in the imprinted polymer. In particular, the authors analyzed the impact on the film density of three ionic liquids as supporting electrolytes in methanol electropolymerization solution, namely 1-hydroxyethyl-3-methylimidazolium hexafluorophosphate ([HOEMIm]PF_6_), 1-hydroxyethyl-3-methylimidazolium tetrafluoroborate ([HOEMIm]BF_4_) and 1-hydroxyethyl-3-methylimidazolium bis((trifluoromethyl)sulfonyl)imide ([HOEMIm]NTf_2_). The results suggested that, while the number of imprinted sites depended on both cation and anion, the anion size had an impact on the polymerization rate, meaning that when the anion is larger, the film becomes more porous, allowing a greater diffusion of the redox probe used in the detection step, that occurred more favorably when compared to conventional supporting electrolytes as TBAP [34].

Herein, we report on the environmentally safe and easy electrosynthesis of a molecularly imprinted polymer from a thiophene-derivative in an ionic liquid, namely 1-butyl-3-methylimidazolium bis(trifluoromethylsulfonyl)imide ((BMIM)TFSI), a room-temperature ionic liquid [35]. Specifically, 2,2′-bis(2,2′-bithiophene-5-yl)-3,3′-bithianaphtene (BT_2_-T_4_) was selected as a functional monomer, whose synthesis has been studied and optimized in our research group [36]. Although the BT_2_-T_4_ molecule was used as a cross-linking agent in the production of molecularly imprinted polymers for the sensing of melamine [33], ATP [37], and 2,4,6-trinitrophenol [38], it has not been exploited as functional monomer before. Tyrosine is used here as a case study template molecule leveraging the reported diastereomeric interactions with the thiophene-derivative functional monomer [39], enabling tyrosine enantiomeric recognition on inherently chiral electrodes obtained by the electropolymerization of a BT_2_-T_4_ enantiomer. Tyrosine is an aromatic, non-polar, non-essential amino acid deriving either from diet or amino acid phenylalanine with significant clinical relevance. Deficiencies in tyrosine hydroxylase lead to a pathophysiology mainly characterized by dystonia and encephalopathy, while tyrosine metabolism deficits, collectively known as tyrosinemia, concern the impossibility of catabolizing the amino acid, giving rise to increased blood levels that affect liver, kidneys and nervous system [40,41]. Additionally, tyrosine supplementation seems to reinforce dopamine production, thus having a role in stress conditions such as major depressive disorder (MDD) or clinical depression [42,43,44,45,46].

Tyrosine, and amino acids in general, is generally detected with sensitive and robust methods, such as HPLC [47], LC-MS/MS [48] and fluorometric assays [49], although the application of such technologies often requires expensive instrumentations and trained personnel, preventing their implementation to the Point-of-Care (PoC) [50]. It is, therefore, fundamental to develop a fast-responding, sensitive and user-friendly device able to detect specific amino acids. In this regard, electrochemical sensors meet this requirement by assuring a rapid, selective and reproducible response to the aminoacid target, as well as the possibility to specifically functionalize the electrode surface and to miniaturize the device itself, allowing an affordable and early diagnosis of patient conditions [51,52,53,54]. For example, Karazan and Roushani achieved the simultaneous detection of tyrosine and ascorbic acid by a glassy carbon electrode functionalized with a dual molecularly imprinted polymer based on o-aminophenol and m-dihydroxy benzene, showing limit of detection values in the nanomolar range, fast and selective sensor responses and adequate recovery estimations in spiked serum sample [55]. Mahdi et al. easily developed an electrochemical sensor based on molecularly imprinted catechol and para-aminophenol for the concomitant detection of tryptophan, dopamine and riboflavin, reporting a linear and selective response towards the analytes with low limit of detection values [56]. Sebastian et al. decorated a disposable screen-printed carbon electrode with a zinc cobaltite 3D reduced graphene oxide (rGO) nanocomposite for L-tryptophan detection, claiming a sensor response in the nanomolar range and excellent recovery percentages in dairy products [57]. Recently, Li and coworkers combined a molecularly imprinted poly(o-phenylenediamine) with multi-walled carbon nanotubes (MWCNTs), chitosan and β-cyclodextrin to produce an electrochemical sensor for the enantiomeric detection of L-tryptophan resulting in a limit of detection of 0.5 μM and a good recovery estimation in spiked milk samples [58]. Given the outstanding electrocatalytic properties of MWNCTs [59,60], such excellent material was further exploited in combination with molecular imprinting technology in the work of Yu et al., where a selective electrochemical sensor was constructed for the nanomolar detection of glutamic acid and its sensing in pig serum samples, results that were additionally corroborated by HPLC analysis [61].

The present work combines the advantages of electrochemical sensing with a green method for the development of a BT_2_-T_4_-based MIP for tyrosine. The greenness of the proposed approach relies not only on the replacement of organic solvents with IL but also on the use of a few microliters of the polymerization solution drop-casted on screen-printed electrodes (SPEs), devices that integrate working electrode, pseudoreference electrode and auxiliary electrode on the same platform. Along with the very low volumes required [62] and low cost, applicability on real-time and on-site sensing [63] are additional benefits, all contributing to the sustainability of the proposed approach, along with its possible use in PoC contexts. 

The developed MIP exhibits good performance in tyrosine amperometric detection in terms of linear response and limit of detection, as well as selectivity, stability, and reproducibility. Lastly, the sensor was applied for quantitative tyrosine detection in real samples, resulting in a satisfactory recovery estimation in human plasma. The overall results showed that our MIP development strategy meets the requirements of the green chemistry approach as well as the potentiality of the as-produced sensor for tyrosine detection in a real sample.

## 2. Results and Discussion

### 2.1. MIP Electrosynthesis

**Figure 1:**
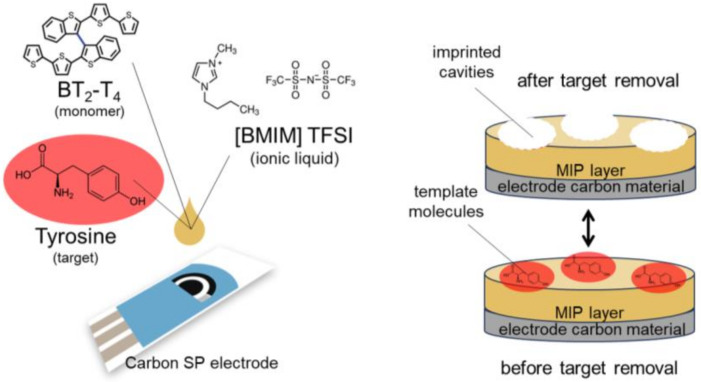
Schematic representation of the as-proposed MIP-based sensor for tyrosine.

The polymerization solution was prepared by dissolving 10 mM racemic BT_2_-T_4_ and 5 mM tyrosine in (BMIM) TFSI, obtaining a clear solution after a few minutes in an ultrasonic bath at room temperature, highlighting the great potentiality of the ionic liquid as solvent. It is noted that due to the lack of BT_2_-T_4_ solubility in water, as commonly observed for thiophene-derivatives, only organic solvents could be used for the preparation of polymerization mixture, not only having an impact on health and environment but also preventing the use of screen-printed electrodes that are typically suitable for aqueous solutions only. The significant solubility shown in ionic liquids represents a key element for replacing organic solvents, enabling, at the same time, the use of low-volume screen-printed cells. As schematically reported in Figure 1, 20 µL of the polymerization solution was dropped on the C-SPE covering all electrodes, thus allowing polymer electrochemical deposition on the electrode surface. The final step of MIP production was achieved with the template removal in CH_3_OH/H_2_O/CH_3_COOH solution, leading to the formation of imprinted cavities.

**Figure 2:**
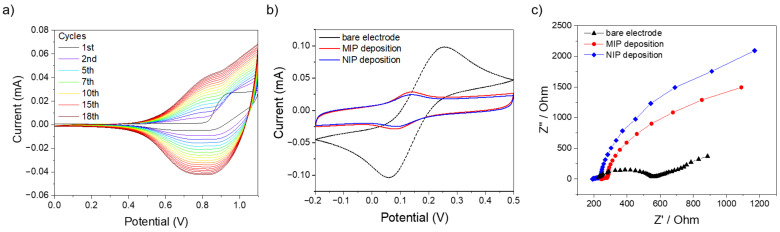
(**a**) Cyclic voltammetry of BT_2_-T_4_ electropolymerization. (**b**) CV curves of bare electrode, MIP and NIP deposition in [Fe(CN)_6_]^3−/4−^. (**c**) EIS curves of bare electrode, MIP and NIP deposition in [Fe(CN)_6_]^3−/4−^.

Figure 2a shows the cyclic voltammetry curve for the electropolymerization of BT_2_-T_4_. The polymer formation started in the first cycle with the monomer oxidation at around 0.9 V with a current value of 40 µA. By increasing the number of cycles, a broad peak appeared in both direct and inverse scans, with progressively increasing currents, suggesting the formation of a conducting polymeric film. This is confirmed by MIP film electrochemical behavior monitored by CV and EIS measurements in [Fe(CN)_6_]^3−/4−^ solution, reported in Figure 2b,c, respectively. In particular, the presence of probe redox peaks in the CV profile clearly reveals the conductivity of the polymeric film. The same result is observed on both MIP and NIP films, with only a slight increase of redox current on the former—along with a minor impedance decrease—possibly ascribed to the presence of templated cavities promoting the probe’s direct access to the electrode surface. 

### 2.2. Optimization of Detection Parameters

**Figure 3:**
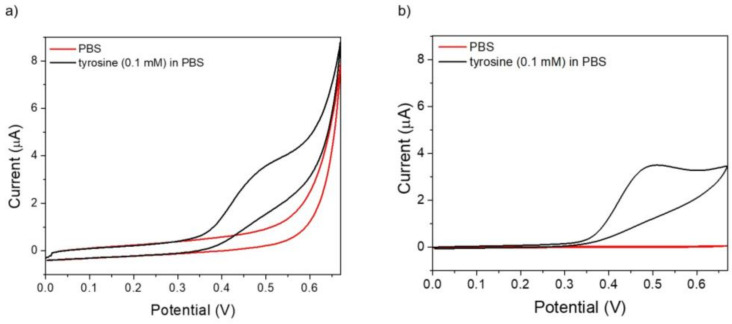
Electroactivity of 0.1 mM tyrosine in PBS on (**a**) MIP electrode and (**b**) bare screen-printed carbon electrode. Potential range: 0–0.67 V, scan rate: 50 mV/s.

Tyrosine oxidation potential was evaluated by cyclic voltammetry in PBS on an MIP-modified electrode and on a bare electrode as a reference. An anodic peak at about 0.5 V (Figure 3a) was recorded on an MIP electrode, ascribed to tyrosine oxidation in an irreversible reaction process with the loss of two electrons and two protons leading to a quinonic byproduct [64]. A similar tyrosine oxidation potential value is observed on a bare electrode (Figure 3b), with a different CV profile at higher potentials evidently ascribed to MIP film electroactivity, also observed on voltammetry in a buffer. 

**Figure 4:**
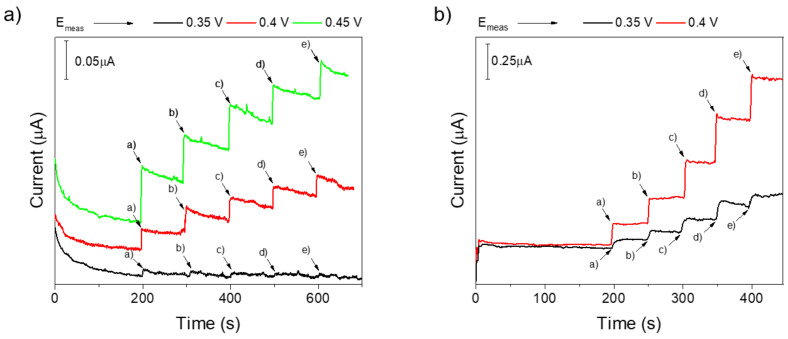
(**a**) Chronoamperometric MIP response towards tyr increasing concentrations (a = 15 μM, b = 30 μM, c = 50 μM, d = 75 μM, e = 100 μM) at constant potential (black: 0.35 V, red: 0.4 V, green: 0.45 V). (**b**) Multiple pulse amperometric response of MIP towards tyr increasing concentrations (a = 15 μM, b = 30 μM, c = 50 μM, d = 75 μM, e = 100 μM) at different measurement potentials (E_meas_, black: 0.35 V, red: 0.4 V).

Moving from this evidence, three different potentials have been tested for tyr 15–100 µM amperometric detection, namely 0.35, 0.4, and 0.45 V, for evaluating the oxidation current at slightly lower potentials, at which the possible interference from other electroactive species could be attenuated. As shown in Figure 4a, at 0.35 V, a slight current response can be appreciated after each tyr addition, while at 0.4 V and 0.45 V, current responses are significantly enhanced, as expected. Nonetheless, particularly at higher potentials, an evident electrode fouling can be observed due to the adsorption of tyr oxidation byproducts, which determines the instability of the recorded current upon each tyr addition.

For these reasons, multiple pulse amperometric detection (MPAD) was tested, with a three-potentials program applied, consisting of a working potential for the analyte oxidation (E_measurement_ (E_meas_)), during which the current is recorded and two additional pulses (E_1_ and E_2_) repeatedly applied to regenerate the electrode surface from adsorbed species [65]. Specifically, based on preliminary amperometric measurements, two working potentials were tested, namely 0.35 V and 0.4 V, not including 0.45 V, due to more favorable co-oxidation of other molecules possibly coexisting with tyr in real matrices. E_1_ was set to 0.5 V, while 0.1 V was chosen as E_2_, with each potential applied for 0.2 s. Collected results are reported in Figure 4b, where it is immediately evident that the condition determining the highest current response consists in E_meas_ of 0.4 V. Interestingly, the effect of the applied pulsed potential program on the current stability suddenly emerges. After each tyrosine addition, the current increase is kept with time, suggesting the absence of adsorbed species limiting the electron transfer to the electrode surface. The application of 0.5 V (E_1_) thus allows complete tyrosine oxidation, whose products are then removed from the electrode surface by the subsequent application of a more cathodic potential (E_2_), which enables the electrode regeneration for further tyr detection. Therefore, 0.4 V was selected as the measurement potential with E_1_ = 0.5 V and E_2_ = 0.1 V.

### 2.3. Tyrosine Amperometric Detection

**Figure 5:**
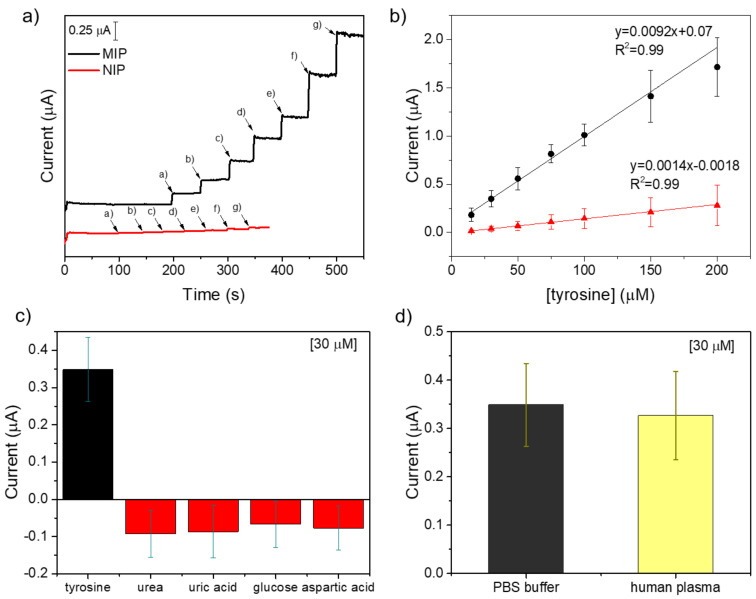
Analytical characterization of the MIP-based sensor for tyr. (**a**) MPAD response of MIP towards tyr increasing concentrations (a = 15 μM, b = 30 μM, c = 50 μM, d = 75 μM, e = 100 μM, f = 150 μM, g = 200 μM) compared with the NIP response. (**b**) Calibration curve of MIP response. (**c**) Selectivity test of MIP response against interfering molecules. (**d**) Amperometric tyr detection in spiked human plasma.

Under the optimized conditions, the MIP sensor response was tested by increasing tyrosine concentrations from 15 to 200 µM (Figure 5a). A linear current increment was recorded, as shown by the calibration curve in Figure 5b, with good linearity (R^2^ = 0.99) in the whole range. The limit of detection (LOD) and the limit of quantification (LOQ) was evaluated as the ratio between the standard deviation of the signal related to blank solutions (solutions without the target) (σ) and the slope (S) of the calibration curve (3.3σ/S and 10σ/S), equal to 1.04 µM and 3.17 μM, respectively. NIP electrode was tested under the same conditions, revealing an almost negligible response at each tyr concentration, demonstrating the high specificity of MIP response. The remarkable specific contribution to sensor response is quantified by the imprinting factor (IF), calculated as the ratio of slope from MIP and NIP calibration curves (IF = S_MIP_/S_NIP_) [66], and resulting as equal to 6.6, marking the noteworthy imprinting effect possibly ascribed also to the effect of ionic liquid [67]. The imprinting factor obtained in this work results higher or comparable with those reported for other MIPs prepared in ionic liquids, for which IF values from 2 to 10 are indicated [68,69,70,71].

Sensor response inter-electrode reproducibility was evaluated by comparing three different MIP electrodes at 75 μM tyr, resulting in an acceptable variability (RSD% = 11.4%). Additionally, the sensor repeatability was measured by three replicates on the same electrode, with RSD% equal to 3.3%, indicating a consistent sensor performance. 

### 2.4. Sensor Selectivity, Stability and Real Sample Analyses

Selectivity against some interfering molecules that could be present in the same tyrosine environment was studied. Current responses recorded to 30 µM tyrosine and equal concentrations of urea, uric acid, glucose, and aspartic acid are plotted in Figure 5c. No oxidation process was observed upon sensor exposure to interfering molecules. However, a slight current drop was observed, probably due to the adsorption of molecules onto the electrode surface. 

The sensor stability was analyzed after 15 days of storage at room temperature in open air without any treatment. Good stability was observed, with sensor response variation of only 3.2% (RSD%, *n* = 3).

Real sample analysis was performed, testing sensor response in human plasma spiked with 30 µM tyrosine upon verifying the absence of sensor response in untreated samples. A satisfactory recovery of 93.6% was obtained (Figure 5d), revealing the sensor’s ability to operate in real samples with negligible matrix effect. 

The performance of the developed MIP-based sensor for tyrosine electrochemical detection results is comparable with some other recent electrochemical sensors reported in the literature, as shown in Table 2 [72,73,74,75]. It should be highlighted that, given the reference values of tyrosine in human organisms, the sensor’s linear range response makes it suitable for physiological and pathological state monitoring, the latter including tyrosine deficiency and excess [76,77,78,79,80,81]. Moreover, in such a comparison with other detection systems, additional beneficial aspects of the developed sensor should be taken into account, including the use of ionic liquid as a green solvent and low-volume electrochemical cell, in compliance with green chemistry guidelines, which should increasingly become cornerstones of research and application fields. 

## 3. Materials and Methods

### 3.1. Reagents and Instruments

All chemicals were of analytical grade and used as received. The working solutions were prepared using ultra-pure water with a conductivity of <0.1 µS/cm. The chemical reagents used included tyrosine, urea, uric acid, glucose, aspartic acid, citrated human plasma, methanol, and human plasma purchased from Sigma Aldrich (St. Louis, MO, USA). 1-Butyl-3-methylimidazolium bis(trifluoromethyl sulfonyl)imide ionic liquid (BMIM) TFSI was provided by Tokyo Chemical Industry (TCI) and stored at room temperature before use. The racemic mixture of 2,2′-bis [2,2′-bithiophene-5-yl]-3,3′-bithianaphthene (BT_2_-T_4_) in powder was produced by the Department of Chemistry at the University of Milan (Italy) and stored at 4 °C until the use. Phosphate buffer solution (PBS) at pH 7 was prepared by dissolution of KH_2_PO_4_ and Na_2_HPO_4_, adjusting the final pH with NaOH 5 M. Disposable screen-printed carbon electrodes (C-SPE), with a carbon working electrode, a silver pseudoreference electrode, and a platinum auxiliary electrode, were used and functionalized with MIP layers. Electrochemical measurements were carried out with PalmSens4 Potentiostat integrated with PSTrace software version 5.6.1608.

### 3.2. Electrosynthesis of the MIP

Ten millimolar racemate BT_2_-T_4_ as a functional monomer and 5 mM of tyrosine as a template were dissolved in ionic liquid (BMIM) TFSI to obtain the polymerization solution. Then, 20 µL of this solution was dropped on the C-SPE. The electrosynthesis of MIP films was carried out via cyclic voltammetry (CV) in the potential range 0–1.1 V at 50 mV/s for 18 cycles, followed by cyclic voltammetry in a monomer-free ionic liquid in the potential range 0–1.1 V at 50 mV/s for 50 cycles to carry away unreacted molecules. To remove the target template, the modified electrode was washed with a solution prepared using methanol, ultra-pure water, and acetic acid (15/55/30, *v/v*) for 2 h under stirring (250 rpm). After each functionalization step, the electrode was rinsed with water and dried with a gentle stream of nitrogen. For comparison, non-imprinted polymers (NIPs) were obtained in the same conditions but using a polymerization solution without the template. Electrochemical characterization of bare screen-printed carbon electrode, as well as MIP and NIP film deposition was performed with 50 mM PBS containing 5 mM [Fe(CN)_6_]^3−/4−^ and 0.1 M KCl as supporting electrolyte by cyclic voltammetry (CV) in the potential range −0.2–0.5 V at 50 mV/s and by electrochemical impedance spectroscopy (EIS) in the frequency range 10.000–0.1 Hz at E_dc_ = 0.15 V and E_ac_ = 0.005 V. 

### 3.3. Electrochemical Measurements

Tyrosine oxidation was evaluated both on bare screen-printed carbon electrode and MIP, employing a target solution (0.1 mM) prepared in 0.1 M PBS via CV in the potential range between 0 and 0.67 V at 50 mV/s for one cycle. Amperometric measurements were performed using a working solution of 0.1 M PBS under stirring (250 rpm) by increasing tyrosine concentrations. The difference between the current recorded after each addition of tyrosine in the electrochemical cell and the background current (ΔI = I_tyr_ − I_background_) was used as an analytical parameter to construct the calibration curve. Each point of the calibration curve refers to the average of three replicates performed on three freshly prepared sensors (*n* = 3). 

## 4. Conclusions

An environmentally safe and sustainable approach was adopted to develop a thiophene-derivative-based molecularly imprinted polymer for amperometric tyrosine detection. The green chemistry guidelines were met using a room-temperature ionic liquid as a green solvent and screen-printed carbon electrodes to reduce the reagent volume. The sensor response resulted linearly in the analytical range of 15–200 µM, with a good detection limit and a great imprinting factor, marking the potentiality of the imprinting process in an ionic liquid as solvent. Additionally, the sensor showed no significant response towards common tyrosine interfering molecules, namely urea, uric acid, glucose, and aspartic acid, as well as stability upon 15 days of storage at room temperature, repeatability, and reproducibility. Finally, the sensor was used for tyrosine detection in spiked human plasma samples, resulting in a satisfactory recovery estimation.

## Figures and Tables

**Table 1:** Common cations and anions constituting ionic liquids.

Cations	Anions
1-alkyl-3-methylimidazolium	halide
1-alkyl-1-methylpyrrolidinium	tetrachloroaluminate
1-alkyl-1-methylpiperidinium	tetrafluoroborate
tetraalkylphosphonium	hexafluorophosphate
tetraalkylammonium	bis(trifluoromethane sulfonyl)imide

**Table 2:** Summary of recent electrochemical sensors for tyrosine detection.

Electrode	LOD (μM)	Linear Range (μM)	Ref.
rGO-MIP/GCE	0.0032	0.01–100	[72]
Cysteic acid/GCE	0.55	1.7–50	[73]
MIP/pTH/Au@ZIF-67/GCE	0.00079	0.01–4	[74]
Unmodified C-SPE	1.15	1–150	[75]
MIP/C-SPE	1.04	15–200	This work

## Data Availability

The data presented in this study are available in the article.
